# Living Donor Liver Transplant Programs in the United States Need to Be Carefully Nurtured Amidst Expanding Use of Perfusion Technology

**DOI:** 10.3390/jcm14072259

**Published:** 2025-03-26

**Authors:** Sorabh Kapoor, Chirag S. Desai

**Affiliations:** Division of Abdominal Transplantation, Department of Surgery, University of North Carolina, Chapel Hill, NC 27599, USA

**Keywords:** living donor liver transplant, normothermic regional perfusion, machine perfusion

## Abstract

Living donor transplantation constitutes a small portion of total transplants in the United States as compared Southeast Asia and Middle East. Recent consensus meeting has identified reluctance on the part of transplant providers and donor financial concerns as the major hindrance in increasing the Living donor liver transplants in US. There is a need to carefully analyze the recent outcome data from across the globe and from large volume North American centers that clearly establishes the benefit of Living donor transplants for both adults and children and reducing wait list mortality. LDLT also provides an opportunity for expanding the indications to offer transplant for indications like colorectal metastasis and intrahepatic cholangiocarcinoma without reducing the number of livers available for traditional indications. Recent expansion of perfusion technology has demonstrated significant increase in utilization of Non heart beating donor livers over the last few years. However, with simultaneous increase in patients being added to the wait list, the wait list mortality and dropouts have been persistently high. In this opinion piece, the authors have looked at the transplant trends in the US in the last few years and advocate for adopting a complementary rather than a singular approach for expansion of LDLT along with new perfusion technologies for increasing the number of liver transplants in the US.

## 1. Introduction

**Figure 1:**
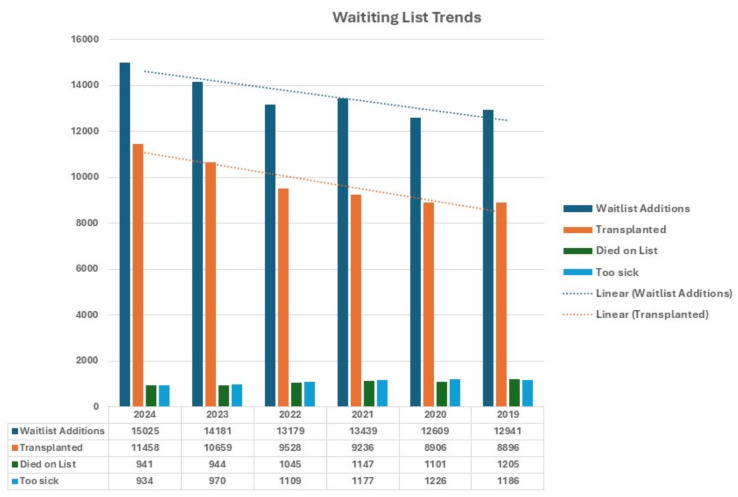
UNOS SRTR data (2019 to 2024) with annual waitlist additions for liver transplantation, total transplants, and waitlist removals by death or illness.

In recent years, there has been a resurgence in the use of Donation after Cardiac Death (DCD) grafts for liver transplantation, predominantly due to the wide utilization of Normothermic Machine Perfusion (NMP) and Normothermic Regional Perfusion (NRP) [1,2,3] (Figure 1). Both machine perfusion and NRP have demonstrated reductions in early allograft dysfunction and biliary complications compared to Standard Cold Storage (SCS). The increasing adoption of these techniques has resulted in more transplants using DCD livers [1,2,3]. The advantages of these devices include portability, physiological assessment, and organ conditioning, which are anticipated to decrease the liver-discard rate [3,4]. Simultaneously, the number of living donor liver transplants seems to have stagnated in the USA [2,3].

## 2. Methods

In this opinion piece, the authors evaluated the OPTN data for liver transplants performed in the USA since 2019 to see how the increasing use of machine perfusion impacts transplant numbers and utilization of DCD livers. OPTN data were evaluated for the impact on patients on waiting list mortality or removal due to worsening medical condition. The findings from recent consensus meetings describing barriers to living donor liver transplantation and recent manuscripts reporting outcomes of LDLT from North America were reviewed. Additionally, we also reviewed the possible impact of these changes on the wider utilization of LDLT in the USA.

## 3. Discussion

### 3.1. Change in Transplant Trends

However, the rapid rise in DCD donations has not narrowed the gap between new patients being listed and transplants performed, with almost 4000 patients remaining on the list and approximately 1000 dying annually [2] (Figure 1). This persistent gap between listing and transplants is partly due to population growth and the rising incidence of MASLD (Metabolic Dysfunction-Associated Steatotic Liver Disease), alcohol-induced liver disease, and the listing of younger patients with acute alcoholic hepatitis. The wider availability of transplants also increases the number of patients who are listed as active at a lower MELD.

### 3.2. Impact of the Use of Newer Perfusion Technology on Transplant Centers

The increasing use of perfusion techniques involves significant costs related to devices, consumables, and personnel, along with infrastructural and resource utilization. Elevated organ acquisition costs are currently borne by the centers as machine perfusion may not be fully reimbursed by different payers. Timely transplants using non-heart-beating donor livers with new perfusion technology can reduce long-term healthcare costs by decreasing waiting times and postoperative stay. However, the upfront costs can be financially challenging for mid-to-small-volume programs and may increase the already existing disparities in centers in underserved areas [5,6]. There have been multiple reports looking at the cost–benefit analysis of perfusion technologies. Most reports look at the economic impact, including the reduction in hospital stay by reducing early allograft dysfunction for marginal grafts, and cost–benefit over the long term by reducing waiting list costs and increasing Quality-Adjusted Life Years (QALYs), while the cost of NRP, including for failed DCD runs, has been included in some analyses [7,8].

The reimbursement is complex and subject to numerous factors, such as the type of insurance, the type of pump, and transport or personnel costs, which may vary for each case. Pioneers in the use of NRP also acknowledge that the acquisition costs will be higher for newer technologies, especially more so for NMP, and when hypothermic pumps may be used post-NRP [9].

The impact of non-progression of a DCD donor for a center is significant, as personnel, transport, and consumable costs are often non-refundable or partially refundable. NMP and NRP will continue to deal with a considerable proportion of donors not progressing, unacceptable biopsies, warm ischemia times, or post-perfusion non-utilizations due to several reasons [10].

There is no doubt that any measures to increase organ use for marginal grafts and reduce allograft dysfunction and ischemic cholangiopathy with expanding use of DCD grafts using NRP, NMP, or when hypothermic pumps become available in the US, will be beneficial to the health system by reducing waitlist and in-hospital costs in the long-term analysis, but the increase in organ acquisition costs are undeniable.

Simultaneously, the ethical concerns voiced by many regarding the reestablishment of circulation and cardiac activity with TA-NRP still exist and have the potential to harm the public’s perception regarding the organ donation process [11,12]. Efforts at reaching a consensus on TA-NRP will require extensive public discussions before widespread adoption across all OPOs.

Furthermore, reliance on third-party pumps confers significant financial burdens and logistical challenges due to procurement scheduling. The data from the last five years show a steady increase in liver transplant numbers, predominantly by a >3-fold increase in DCD liver transplants, that is, however, accompanied by simultaneous increase in patients being listed for transplant and a relatively persistent incidence of patients dying on the waitlist or removed because they became “too ill” [2] (Figure 1).

### 3.3. Benefits of a Viable LDLT Program

Early transplant, especially when using LDLT, is undoubtedly beneficial for outcomes and survival and makes financial sense by reducing costs due to waitlist admissions and procedures [13,14,15,16,17]. Living Donor Liver Transplantation (LDLT) continues to play an important supplementary role in allowing timely transplants for low-acuity patients. Additionally, LDLT often offers timely possibilities for transplantation for newer indications such as expansion of HCC (Hepatocellular Carcinoma) criteria, CLRM (Colorectal Liver Metastasis), or ICC (Intrahepatic Cholangiocarcinoma), as these patients are invariably listed with low clinical MELD scores. The benefits of LDLT and the donor risks and oncological outcomes in these indications will need to be discussed in depth by the transplant and oncology teams with both the donor and recipients.

Modifications in organ allocation aimed at minimizing mortality and newer preservation strategies have not resulted in lowering the average MELD for transplantation significantly over the last five years. The MMAT (Median MELD at Transplant) continues to be 28 across the USA after recent acuity circle implementation [18]. In this scenario, the increasing use of DCD graft supplemented by promoting LDLT numbers offers an opportunity for reducing wait times and waitlist mortality.

### 3.4. Status of LDLT in the USA

**Figure 2:**
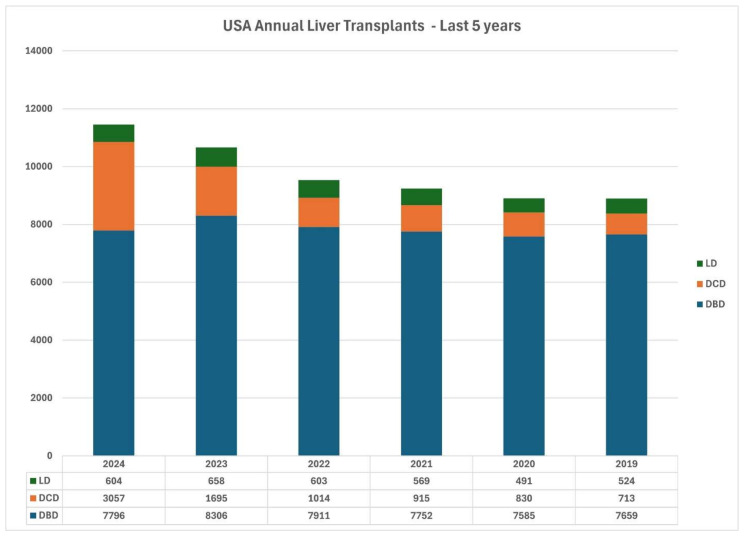
UNOS SRTR data (2019 to 2024) with annual liver transplants by donor type.

Unlike in Asia, where LDLT constitutes most liver transplants, in North America, LDLT accounts for less than 5% of the total. Despite an increase in absolute LDLT numbers from 524 cases in 2019 to 604 in 2024, LDLT only accounts for 5–6% of total liver transplants. The slow growth of LDLT in the USA is concerning, with only 10 centers performing 20 or more LDLTs in the last two years (equivalent to half of the US LDLT volume) [2] (Figure 2).

### 3.5. Pediatric LDLT in the USA

Expanding pediatric LDLT programs can be a viable strategy for increasing living donor transplants. The concerns regarding the donor’s liver remnant can be minimized due to the smaller portion of liver removed for a left lateral graft or a left lobe graft. However, pediatric liver transplants, especially living donor transplants, require significant expertise, not just for the actual surgery but also for expert Intervention Radiology, Hepatology, and Psychology support for dealing with complex pediatric patients [19].

**Figure 3:**
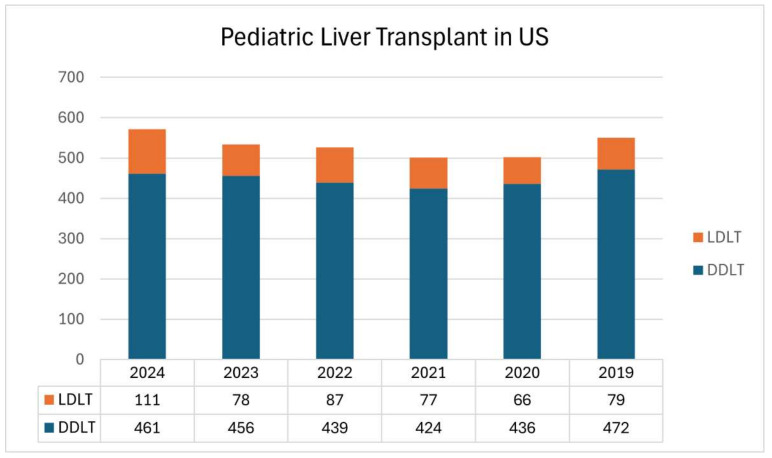
UNOS SRTR data (2019 to 2024) pediatric liver transplants by donor type.

Unfortunately, pediatric living donor liver transplants have not grown significantly in the last few years in the USA (Figure 3). Despite a clear demonstration of the survival benefit, from a large-volume adult and pediatric LDLT center in North America, of having a living donor while a child is on the waiting list, multiple factors, such as lack of expertise and the complexity of managing pediatric patients before and after transplantation, have not allowed increases in the utilization of LDLT beyond a few established pediatric living donor liver transplant centers [19,20].

### 3.6. Can Wider Expansion of DCD Livers in the USA Replicate European Experience?

The utilization of DCD donors in conjunction with perfusion techniques has improved outcomes in parts of Europe; however, waitlist mortality still ranges close to 10% or more [21].

Improved donation and DCD utilization rates have led to minimized wait times in countries like Spain; however, that has not been the case in other European nations [21,22].

Non-utilization rates in the USA and UK are often reported as being disproportionately high compared to other European nations. However, the reported difference in numbers can be partially accounted for by careful analysis of DCD livers that are not regarded as potentially transplantable during the initial DCD assessment in certain nations, compared to the inclusion of all DCD liver offers in the US and UK. Countries similar in population to the UK, like France, Spain, and Switzerland, have initial rule outs that might be responsible for the high DCD utilization rates compared to the US and UK [22].

Hence, the real possible increase in potential donors with increasing utilization rates may reduce but not completely bridge the waitlist mortality despite wider spread of NRP and DCD. Recent data have shown that, despite a threefold increase in DCD transplants in the last two years, the gap between listed and transplant patient could be reduced to some extent but not completely bridged [2] (Figure 1).

### 3.7. Why LDLT Continues to Stagnate in the USA

Multiple factors account for the limited LDLT numbers in the USA, including the transplant program (and providers’) reluctance to offer LDLT to patients due to earlier inferior results from the USA (recipient selection had a significant role in this) and instances of highly publicized donor deaths [23]. The recent rise in DCD transplants and pervasive ethical concerns on the part of the transplant community about donor risk and complications have prevented an evidence-based acknowledgment of current data on the benefits of LDLT in terms of outcomes, safety, and cost–benefit, as demonstrated in select large volume North American Centers, including US centers [13,14,15,16,17]. The direct financial advantage compared to expenditure on machine or regional perfusion is also directly available to the transplant center along with reduced overall healthcare expenses based on lower waitlist costs and hospital stay.

There are multiple challenges for starting an LDLT program in the USA. Adhering to federal and state regulations, OPTN, the Centers for Medicare & Medicaid Services (CMS), etc., is crucial. Surgeons and programs need to complete a finite number of cases in a fixed time for full accreditation. Apart from recruiting or training a donor surgeon who can serve as a lead living donor surgeon, there are no recurring costs per case, as most liver programs already have the required infrastructure and personnel in the team. Addressing ethical concerns regarding donor risks with providers, patients, and donors and ensuring informed consent is critical. Well-defined protocols for recipient and donor evaluation, including advanced radiology services, post-surgical management, and risk management are necessary to ensure optimal outcomes. Experienced donor surgeons (US- or overseas-trained) can help bypass the learning curve for donors with multiple vessels and ducts. Raising awareness about the benefits and risks of living donor liver transplantation remains the most challenging aspect for program growth. Growth of pediatric living donor transplantation can be achieved by expanding collaboration with established national and international pediatric living donor programs that can provide guidance in mentorship to adult-specific LDLT programs or programs interested in starting pediatric LDLT alone.

The American Society of Transplantation recently held a consensus meeting to evaluate barriers to the slow growth of LDLT in the USA and identified key issues that need to be addressed to expand LDLT numbers [24,25]. The working group identified three major areas of concern—(1) challenges with provider, center, and patient (and potential donors) attitudes towards LDLT, (2) variable donor and recipient selection with often very restrictive selection criteria based on perception and old outcome data rather than newer real-world data, and (3) lack of adequate country-specific information regarding long-term donor outcomes. Although a majority of LDLT and non-LDLT centers accepted the benefit of LDLT, less than one-third of respondents in a survey considered LDLT as the first option [25]. The presence of financial barriers related to donor loss of wages, and expenses on travel, for evaluation were the top priority in the consensus meeting, followed by concerns regarding donor risk [24]. The involvement of multiple stakeholders and desire to increase LDLT is encouraging; however, the expansion in small-volume or new centers continues to be constrained by lack of resources and expertise, or provider reluctance. Apart from a limited number of centers, there is still hesitancy to offer LDLT as a routine option. The barriers to expanding LDLT identified by the consensus meeting are not new. Apart from a few centers and enthusiastic providers, efforts by the Transplant Professional Society have not been successful. The increased use of perfusion technologies and the optimism around increased DCD transplants have the potential to, unfortunately, negatively impact the efforts by AST and ASTS to expand LDLT numbers in the USA, especially for sceptics and people on the fence. It is important to remember that the goal is not to vehemently support one strategy or technology, but rather to embrace all avenues for expanding transplant numbers and minimize waiting list mortality and dropouts.

### 3.8. The Way Forward

AST and ASTS must effectively deal with the challenges identified in the consensus meeting by standardizing pre-donation information, use of recent outcome data substantiating the benefit of LDLT for survival, and advocacy for identifying ways of mitigating donor financial implications at the state and federal level, such as reimbursement for lost wages, covering travel and non-medical evaluation expenses. The adoption of “Donor Shield”, like that offered to NKR kidney donors, by all LDLT centers may reduce the financial burden on living donors and encourage more donors to volunteer. Currently, only ten centers in the USA offer the Donor Shield for living liver donors. Universal expansion of the program that will be financed through recipient insurance or federal/state funding, rather than putting the burden on the transplant center, can help in tackling a major identified barrier to the expansion of LDLT [24,26].

Finally, any personal misgivings or prejudices or lack of expertise should not result in a recipient getting deprived of the opportunity to consider a living donor transplant. With expanding indications and waitlists, expanding all options and technologies for timely transplants is important. The argument that expanded criteria for deceased donor grafts with modern preservation will eliminate the need for living donor transplants has not been proven by persistent waitlists in both the USA and Europe, except for certain parts of Europe [21,22]. There is an absence of FDA-approved hypothermic perfusion machines in the USA, outside of trials, and a reliance on expensive NMP options, along with the financial costs of longer distances from donor centers compared to smaller European nations, often requiring air transport [6,9,10]. Newer perfusion devices and use of NRP will, no doubt, help in further increasing the transplant numbers, but will have to keep pace with the simultaneous population rise and waitlist numbers.

## 4. Conclusions

Contemporary liver transplant centers must be ready to optimally utilize LDLT and modern preservation and perfusion techniques to increase transplant numbers, improve outcomes, reduce wait times and mortality, and provide opportunities for transplantation for newer or expanded indications.

## Abbreviations

MASLD	Metabolic Dysfunction-Associated Steatotic Liver Disease
DCD	Donation after Cardiac Death
DBD	Donation after Brain Death
NMP	Normothermic Machine Perfusion
HMP	Hypothermic Machine Perfusion
NRP	Normothermic Regional Perfusion
LDLT	Living Donor Liver Transplantation
MELD	Model for End Stage Liver Disease
UNOS	United Network for Organ Sharing
CRLM	Colorectal Liver metastasis
HCC	Hepatocellular Carcinoma
ICC	Intrahepatic Cholangiocarcinoma
MMAT	Median Meld at Transplantation
QALY	Quality Adjusted Life Years
